# BSAC Vanguard Series: Why culture matters to tackle antibiotic resistance

**DOI:** 10.1093/jac/dkac077

**Published:** 2022-04-06

**Authors:** Esmita Charani

**Affiliations:** 1 National Institute for Health Research Health Protection Research Unit in Healthcare Associated Infections and Antimicrobial Resistance, Imperial College London, UK; 2 Division of Infectious Diseases & HIV Medicine, Department of Medicine, University of Cape Town, South Africa; 3 Department of Infection Control and Epidemiology, Amrita Institute of Medical Science, Amrita Vishwa Vidyapeetham, Kochi (Kerala), India

## Abstract

Research has demonstrated that antibiotic prescribing and use are social processes. Despite the availability of guidelines and policies for optimized use, many challenges remain. Whilst much of the research in antimicrobial resistance is focused on new drugs, the socio-cultural and socio-economic drivers for infections and antibiotic use are also important considerations. Context-specific solutions that are co-developed with end users are needed if we are to optimize the use of existing and new antibiotics. The threat of antimicrobial resistance is not subject to geographical boundaries, and to truly be effective, interventions need to have the potential to be scaled to different settings. The inequities in funding, knowledge generation, ownership and transfer between the global North and South must be acknowledged and eradicated. Striking a balance in funding and equity requires in-country capacity building for: (i) delivering sustainable research; (ii) assuring equitable representation in research outputs; and (iii) supporting career progression of researchers through further funding, to support the generation of locally owned knowledge that contributes to optimized healthcare systems and translation into clinical practice.

## Introduction

Antibiotic prescribing and use are complex processes under the influence of socio-economic and socio-cultural drivers. Human beings have a dynamic and evolving relationship with antibiotics at the biological and sociological level. Biologically, the emergence of antibiotic resistance is potentiated by exposure to antibiotics, through their consumption by humans and animals, and through the environment (e.g. agricultural use and human, animal and manufacturing waste). In the last 30 years, there have been increasing efforts to optimize antibiotic use in human populations and also, as part of a One Health approach, in farming, fisheries, agriculture and the environment. Following WHO guidelines, every country is encouraged to have a National Action Plan that identifies specific objectives for optimizing antibiotic use in humans, and across a One Health agenda. These efforts are supported by a growing body of evidence indicating where gains have been made and where further work is needed. Despite these efforts, suboptimal antibiotic use remains a major concern.

## The social drivers for antibiotic resistance

Discovered in 1928 and mass-produced during the Second World War, antibiotics were touted as wonder drugs that were going to cure heretofore incurable and deadly diseases. Though resistance to them developed very rapidly, the original aura of these ‘wonder drugs’ has not been tarnished in the collective psyche. The overreliance on and false confidence in antibiotics to always save the day remains. Despite the growing evidence and the efforts of scientists, clinicians, and campaigners, the overarching sentiment is that there will be more antibiotics developed and the risk of using existing antibiotics to treat the present patient is lower than any future harm associated with their use. This is a fallacy, as the antibiotic pipeline is not as free-flowing as anticipated, with little incentive for big pharma to invest in antibiotic drug discovery and development. Despite this, the call for more research and development of new drugs is the loudest voice in the antibiotic resistance research field, with much of the initial drug discovery research conducted using public funding through research grants. Though a necessary investment, this alone will not solve the problem of antibiotic resistance. Unless we understand the sociocultural and behavioural drivers for antibiotic use and develop contextually fit, equitable and sustainable strategies to address them, no amount of new antibiotics will cut the tide of emerging antibiotic resistance and its spread through populations.

Human beings operate within social norms and rules that moderate behaviours. Healthcare is a microcosm of human cultures and behaviours, complete with its own tribes, social norms, and microcultures. Research has demonstrated that hierarchies exist within healthcare across professional divides, specialism, and levels of seniority, and they influence antibiotic use and prescribing, despite evidence-based recommendations. One of the failures of our work to date has been to not recognize this when developing strategies to influence antibiotic-prescribing practices. Successful strategies will only work if we understand who the opinion leaders are within specialities, professions, and organizations. Furthermore, we need to understand that clinicians like to practice autonomously and value their own expertise and experience and that of colleagues they consider to be their peers. To influence behaviours, diplomacy is as critical as having a robust evidence base to support one’s arguments. We need to co-develop strategies with peers from the specialities, organizations and professions whose antibiotic-prescribing behaviours we want to change and or optimize. We need to understand the language of risk that will resonate with the target audience. It may not always be sufficient (or even appropriate) to talk about the threat of antibiotic resistance, which is a negative message. First, we must identify what risks and outcomes matter to our colleagues and how the threat of antibiotic resistance would influence these risks and outcomes. That is where the conversation about antibiotic resistance should always start.

## Achieving equity in global health is central to tackling antibiotic resistance

When developing solutions to tackle antibiotic resistance, we often overlook the contextual needs, behavioural drivers, and the stakeholders who need to be involved. This is critical, particularly in collaborations between high-income countries (HICs) and low- and middle-income countries (LMICs). The threat of antibiotic resistance is greatest where there are fewest resources. Solutions invented with research funding in HICs cannot be ‘airlifted’ and applied in LMICs. What is needed is a thorough and thoughtful consideration of the resource limitations and needs in each setting. This means fostering more-equitable research partnerships between HICs and LMICs, built on mutual trust and respect, to enable bilateral learning and knowledge exchange. Infectious disease and antibiotic resistance do not recognize or respect cultural or geographic boundaries. To truly have a chance of stemming the tide of antibiotic resistance, we also need to break through the cultural and geographic boundaries that are influencing the flow of resources and knowledge between the global North and the global South.

Internationally, policymakers, funders and organizations with a stake in global health need to develop expectations and targets for change that reflect the resource limitations of different countries. Funders need to identify strategies for more equitable distribution of research money between HICs and LMICs – currently, over 70% of global health research funding remains in HICs. This requires a shift not only in platitudes and pledging to greater diversity and equity reports, but a seismic change in how we fund research. Equity in funding means giving more resources to LMICs to be able to match what is available in HICs. Achieving the needed balance in funding and equity is dependent on building capacity and supporting in-country researchers to: (i) deliver sustainable research; (ii) have equitable representation in research outputs; (iii) track follow-on career progression through further funding, setting up research teams and emerging leaders. These are measurable targets, which funders can build into a framework for evaluating equity. The above indicators can facilitate the development of a framework for equitable partnerships in global health and antibiotic resistance research. Furthermore, we as clinicians, researchers, and advocates in global health and antibiotic research should advocate for funders to commit to investing at least 50% of all publicly funded research over the next 5 years to equitable research partnerships. To champion this approach there needs to be greater representation of global South scientists, experts, and researchers in (i) identifying research priorities and gaps in global health and (ii) co-developing and leading funding calls and panels. This is where every one of us has a role to play, by speaking up for those not currently represented and making room for them by sharing our platform.

## References

[1] Charani E , McKee M, Ahmad R et al Optimising antimicrobial use in humans – review of current evidence and an interdisciplinary consensus on key priorities for research. Lancet Reg Health Eur 2021; 7: 100161. 34557847 10.1016/j.lanepe.2021.100161 PMC8454847

[2] Charani E , Mendelson M, Ashiru-Oredope D et al Navigating sociocultural disparities in relation to infection and antibiotic resistance—the need for an intersectional approach. JAC-Antimicrob Resist 2021; 3: dlab123 10.1093/jacamr/dlab123. PMC8485076 34604747

[3] Morton B , Verceuil A, Masekela R et al Consensus statement on measures to promote equitable authorship in the publication of research from international partnerships. Anaesthesia 2022; 77; 264–76. 34647323 10.1111/anae.15597 PMC9293237

